# Effects of Invertebrate Iridescent Virus 6 in *Phyllophaga vandinei* and Its Potential as a Biocontrol Delivery System

**DOI:** 10.1673/031.011.0144

**Published:** 2011-04-08

**Authors:** DA Jenkins, WB Hunter, R Goenaga

**Affiliations:** ^1^ USDA-ARS, Tropical Agriculture Research Station, 2200 Ave. P.A. Campos, Ste. 201, Mayaguez, PR 00680.; ^2^ USDA-ARS, U.S. Horticultural Research Laboratory, 2001 South Rock Rd., Ft. Pierce, FL 34945.

**Keywords:** beetle, biological insecticide, *Chilo* iridescent virus, CIV, entomopathogen, IIV6, injection, insect virus, virus delivery system

## Abstract

Invertebrate iridescent virus 6 (IIV6) was determined to cause infection in *Phyllophaga vandinei* Smyth (Coleoptera: Scarabaeidae) through a range of modes of transmissions. This is the first evidence of IIV6 infection in *P. vandinei* that caused both patent and sub-lethal infections in larvae and adults. Mortality rates were determined to be ∼30% when virus inoculum was injected into larvae or adults. Adults injected with virus showed dramatically altered behavior; injected beetles were not observed feeding or mating compared with adults injected with buffer or adults that were not injected. Tissue collected from infected adults resulted in infection when injected into healthy adults, as confirmed with PCR. PCR also confirmed that frass of infected larvae and adults contained virus, and when reconstituted frass from infected individuals was injected into healthy adults or larvae they become infected. Healthy adults could be infected by coming into contact with soil or plant material that had been exposed to infected adults as much as two weeks prior to introduction of nonvirus exposed adults. Although relatively low mortality resulted when adults or larvae were injected with the virus, the demonstration of horizontal transmission, potentially through frass of infected individuals, identifies a mode of transmission that may be exploited as a potential management tool to reduce *P. vandinei*.

## Introduction

Populations of insects are often infected by pathogens that are not lethal and/or do not result in any detectable symptoms in the host. The cabbage moth, *Mamestra brassicae*, was revealed *via* molecular screening to be infected with a virus producing no detectable symptoms (Hughes et al. 1993; Burden et al. 2003). Pathogens that do not result in high mortality of the target insect pest are often overlooked as ineffective biocontrol agents. However, in many cases, sublethal effects of the pathogen may reduce the damage caused by the insect. For instance, sublethal infections by pathogens in the Indian meal moth, *Plodia interpunctella*, affect the development, fecundity, and population dynamics of the insect (Boots and Begon 1994; Sait et al. 1994). More importantly, sublethal infections may provide a ‘delivery system’ of a pathogen that has been designed to increase mortality of the pest or otherwise alleviate the damage this insect causes. The use of sublethal infections as a ‘delivery system’ has been suggested as the focus in the development of highly specific biological control agents (Williams 1998).

*Phyllophaga vandinei* Smyth (Coleoptera: Scarabaeidae) is an important pest of tropical fruit trees on the island of Puerto Rico. The polyphagous and nocturnal adults are defoliators and the larvae can cause severe damage to seedlings by feeding on the roots (Wolcott 1948). The adults emerge at the beginning of the rainy season in April or May to mate and feed on a wide variety of hosts. They continue to feed throughout the summer and, in some locations, a second population peak appears in August-October (Jenkins and Goenaga 2008). *Phyllophaga vandinei* was a significant pest of sugar cane, *Saccharum* *officinarum* L. (Poaceae), when sugar cane was cultivated on the island and researchers tried numerous methods to control *P. vandinei*. In the 1920's the cane toad, *Bufo marinus*, was introduced as a measure of control and it is reported that within a few years the cane toad had dramatically decreased numbers of *Phyllophaga* spp. on the island (Wolcott 1948). However, now the cane toad and *P. vandinei* coexist, both in abundant numbers and, although, based on stomach content of toads, the cane toad appears to consume many *Phyllophaga* adults, *Phyllophaga* populations remain high. Endemic to Puerto Rico, *P. vandinei* had a variety of native predators and parasitoids associated with it. These included flies in the family Bombyliidae and Tachinidae, and the larvae of *Pyrophorus luminosus* (Wolcott 1948), but we have seen no evidence that these parasitoids and predators are having any impact on *Phyllophaga* populations (Jenkins, unpublished data). It is possible that the introduction of the cane toad so reduced *Phyllophaga* populations that many key predators and parasitoids have not recovered, though *Py. luminosus* remains common (Jenkins, unpublished data). Oddly, although there are several species of Scoliidae and Tiphiidae in Puerto Rico, historically these have always been found in very low numbers and are very rarely associated with *P. vandinei* (Wolcott 1948) and this continues to be the case (Jenkins, unpublished data). Assays conducted on several entomopathogenic nematode species against the larvae of *P*. *vandinei*, have shown these to be ineffective so far (Jenkins, unpublished data). Biological control for this important pest is lacking.

Members of the iridoviridae have been recorded from a variety of Poikilothermic animals. Patent infections by iridoviruses are fatal and usually easily recognized by the blue opalescence of the cadaver as a result of paracrystalline arrays of the virus in the tissue. However, in many cases, patent infection occurs in a relatively low proportion of individuals (see Williams 2008). Surveys of patent infection in blackfly species (Diptera: Simuliidae) have typically observed less than 10% of individuals infected (based on cadaver opalescence) (Weiser 1968; Batson et al. 1976; Takaoka 1980; Avery and Bauer 1984; Batson 1986; Erlandson and Mason 1990; Williams and Cory 1993). The use of sensitive bioassays for invertebrate iridescent virus (IIV) infection, including PCR and DNA-DNA hybridization, reveal that a large number of individuals in a population may have covert, non-lethal infections (Ward and Kalmakoff 1991; Williams 1993, 1995; 1996; Marina et al. 1999; Hernandez et al. 2000). The invertebrate iridescent virus 6 (IIV6), synonymous with *Chilo* iridescent virus (CIV) (*Iridoviridae: Iridovirus*), has an icosahedral symmetry with a particle diameter of 120–130nm containing a single copy linear dsDNA genome 212 kbp in length (Jakob et al. 2001). IIV6 was isolated from the rice stem borer, *Chilo suppressalis* (Fukaya and Nasu 1966). Iridoviruses have been reported to infect coleopteran species including the scarab, *Sericesthis pruinosa* (Day and Mercer 1964); Diaprepes root weevil, *Diaprepes abbreviatus* (Hunter et al. 2003, Hunter and Lapointe 2003); and boll weevils, *Anthonomus grandis* (McLaughlin et al. 1972). The objective was to determine the effects of introducing an invertebrate iridescent virus into *P. vandinei* adults.

## Materials and Methods

### Source of *Phyllophaga vandinei* adults and larvae

Adult *P. vandinei* were collected from the foliage of numerous plants on the premises of the USDA-ARS Tropical Agriculture Research Station in Mayaguez, PR. Adults were then placed in screen cages (55 cm × 55 cm × 55 cm) containing moistened vermiculite to a depth of 10 cm. The cages also contained three seedlings of *Manilkara zapota* (L.) P. Royen (Ericales: Sapotaceae). Larvae of *P. vandinei* were obtained from soil in a barren field at the USDA-ARS Experiment Station in Isabela, PR. Larvae were maintained in Solo cups containing diet for *Diaprepes abbreviatus* L. (Coleoptera: Curculionidae) (Product No. F1675, Bio-Serv, www.bioserv.com) as previously described (Beavers 1982).

### Virus Source

An isolate of IIV-6 was obtained from Dr. J. Kalmakoff, University of Otago, Dunedin, New Zealand, through Joel Funk, USDA-ARS (Western Cotton Research Laboratory, 4135 E. Broadway Rd., Phoenix, AZ 85040). The virus was maintained through serial passage into 10 wk old *D. abbreviatus* (Hunter et al. 2003) reared as in Lapointe and Shapiro (1999). Infected larvae were confirmed by PCR and visual inspection of virus pellet harvested 10 d post injection. Virus was then purified using differential centrifugation after Marina et al. (1999). Purified virus was resuspended in 0.1M Tris buffer, pH 7.02, and used, or stored at — 20°C.

### Virus Inoculations

Adults or larvae were exposed to IIV6 by injection, or placement in cages that had contained infected adults for at least two weeks. Control *P. vandinei* were treated in the same fashion, but were inoculated with buffer. Inoculated *P. vandinei* were then assayed for presence of virus using PCR. Injections were done with a plastic 25 µl syringe and a 30 1/2 gauge needle. Insects were injected on the right lateral side of the abdomen, approximately 1/4 the distance of the body length from the anus, with ∼4 µl of either Tris buffer or purified virus suspension. *P. vandinei* were then tested by PCR at least 15 days post treatment. Inoculum not stated as ‘purified virus’ means inoculum that was made from the homogenates of three entire (including the integument) IIV6-infected *D. abbreviatus* larvae, or one entire IIV6-infected *P. vandinei* larva used at least 30 days after infection and virus positive as determined by PCR. The infected *P. vandinei* were homogenized in 3 ml of PBS 1X, pH 7.2, with a ceramic pestle and acid-washed micro glass beads in a ceramic mortar. The homogenate was then centrifuged for 1 min at 14,000 rpm. The supernatant was transferred to a clean 2 ml vial and the pellet was processed for DNA extraction (AquaPure Genomic DNA Isolation Kit, BioRad, www.bio-rad.com). The supernatant was centrifuged again for 2 min at 14,000 rpm and transferred to a clean 2 ml tube. The supernatant volume was increased with the addition of 0.5–1 ml of 0.1 M Tris buffer, pH 7.02. The supernatant suspension was then forced through a 0.45 µm membrane syringe filter. Larvae were inoculated by injection with ∼4 µl of the sterile filtrate and later tested by PCR at least 15 days post injection. Controls were injected with ∼4 µl of syringe sterilized 0.1M Tris buffer, pH 7.02.

To assess mortality caused by infection with IIV6, 10 adult male and 10 adult female *P. vandinei* were injected with the virus, injected with buffer (as described above), or did not receive any injections. Treatments were replicated 5 times. Mortality was assessed 10 days post injection and infection was confirmed using PCR. Analysis of variance (PROC GLM) and Student-Newman-Keuls multiple range test (SAS 2003) were used to analyze the effect of the virus on adult mortality. The distribution of mortality among the sexes was analyzed for departure from equal females and males dying of the virus using a χ^2^ analysis.

### Effect of IIV6 infection on behavior of *Phyllophaga vandinei*

Adult *Phyllophaga vandinei* (30 of each sex) were injected with the IIV6 virus, injected with buffer, or were left untreated. Each group of adults receiving the same treatment was placed in a screen cage (55 cm × 55 cm × 55 cm) with moistened vermiculite to depth of 10 cm and 3 seedling *M. zapota* trees. Cages were stored in a greenhouse on the premises of the Tropical Agriculture Research Station in Mayaguez, PR (25–30° C). The cages were inspected using a flashlight between the hours of 19:30 and 20:30 pm at 24 hours, 48 hours, 90 hours, 174 hours, 222 hours, and 294 hours post injecting and the number of adults on the foliage and number of pairs mating were recorded. Preliminary trials indicated that light exposure did not noticeably disturb mating or feeding behaviour; adults exposed to light continued mating or feeding as long as adults that remained in the dark or were exposed to red filter light. Cadavers were returned to the lab and infection with IIV6 was confirmed using PCR. At the end of one month foliage was removed from all of the seedlings in each cage and the area of foliage removed was calculated by tracing the leaf on graph paper and calculating the area of the whole leaf and the area of consumed foliage. In some cases, where only a stem or a midrib vein remained, it was necessary to estimate the area removed based on an average area of 10 whole leaves from a plant. Each treatment was replicated 5 times. The mean percentage of leaf area removed was compared among the treatments using analysis of variance (PROC GLM) and Student-Newman-Keuls multiple range test (SAS 2003). Because the number of active adults was measured on several occasions, alpha levels were adjusted for each date according to Holm (1979).

### Horizontal transmission

Twenty *P. vandinei* adults (10 males and 10 females) injected with IIV6 from infected *P. vandinei* were placed in a cage (55 cm × 55 cm × 55 cm) containing moistened vermiculite (depth of 10 cm) and 3 seedlings of *M. zapota*. Adults remained in the cage for 2 weeks, after which period all living adults and cadavers were removed from the cage. The cage was left empty for a period of 2 weeks, after which time 20 healthy *P. vandinei* (10 male and 10 female) adults were placed in the cage. After 2 weeks, all living adults and cadavers were removed and infection was confirmed using PCR. This was replicated on 3 occasions.

Frass from a single adult *P. vandinei* was rehydrated with 100 µl of PBS buffer, pH 7.2, to detect IIV6 in the frass of *P. vandinei*. A total of 15 different samples from adults and 12 different samples from larvae 20 days post virus exposure were tested with PCR analysis. Reconstituted frass from a single infected individual was injected into adult *P. vandinei* according to the methods outlined in the virus inoculations section. Successful infection was confirmed by PCR.

### PCR Analysis and DNA Sequencing

Consensus primers were designed for PCR/sequencing based on a conserved region within the capsid protein gene from 3 insect iridoviruses: IIV1, IIV6, IIV22, (GeneBank accession no. M33542; M99395; M32799 respectively) (Webby and Kalmakoff, 1998). Amplification by PCR was conducted with consensus primers: P1FOR (5′ ACYTCWGGKTTYATCGATATCGCCACT 3′) and P2REV (5′ TTRATWGCATGAGAGAARCGAATATC 3′), corresponding to IIV6 major capsid protein nucleotide positions 679–705 and 1548–1573, respectively (synthesized by Invitrogen, Carlsbad, CA, USA). The PCR mix was 1 µl of DNA, 2 µl of primers (50 µM each), 3 µl of 25 mM-MgCl_2_, 45 µl of Platinum® PCR Supermix (Invitrogen, www.invitrogen.com). Cycles were run in an automated Peltier Thermal Cycler, (PTC 200, www.peltier.com). The amplification protocol was: denature at 95° C for 10 min, at 94° C for 2 min, at 41° C for 2 min, and at 72° C for 5 min. Then 30 cycles of: denaturing for 1 min at 94° C, annealing at 41° C for 1 min, elongation at 72° C for 3 min, with a final cycle at 94° C for 3 min, at 41° C for 1 min, at 72° C for 5 min, and hold at 4° C. A 20 µl sample of each reaction mixture was fractionated by electrophoresis in a 1 % agarose gel in TAE 1X buffer and the fragments stained with ethidium bromide. The gel purified 893 bp DNA fragment was sequenced from 3 virus positive larvae and 3 adults, with an ABI Prism 310 genetic analyzer (Applied Biosystems, www.appliedbiosystems.com) using the Dye Deoxyterminator-Tag cycle sequencing technique, as per instructions (Applied Biosystems).

## Results

### Infection of *Phyllophaga vandinei* by IIV6

Adult *P. vandinei* injected with IIV6 showed significantly increased mortality (mean = 31% ± 4%) compared to adults injected with buffer (mean = 8% ± 3%) or adults that did not receive any injections (mean = 7% ± 3%). Most adults (72 %) injected with the virus were confirmed to have the virus using PCR. Of the subset of adults receiving an injection of IIV6 that did not test positive, the vast majority (93 %) died within two days after receiving the injection. None of the injected beetles that died within two days after injection (26 out of 100) tested positive for IIV6 infection using PCR. χ^2^ analyses indicated that distribution of mortality among the sexes (17 males and 14 females out of 100 total adults) did not significantly depart from equal females and males dying (χ^2^ = 1.055; df = 2; *P* = 0.59).

### Effects of IIV6 infection on adult *Phyllophaga vandinei* behavior

No adults injected with IIV6 were ever observed mating, whereas adults injected with buffer (22% ± 3% of total number of caged adults) or adults that received no injections (18% ± 3% of total number of caged adults) were observed mating. Significantly fewer adults that had been injected with IIV6 were observed feeding or aggregating on plant material than adults injected with buffer or which did not received injections. Seedlings of *M. zapota* in cages containing adult *P. vandinei* that had been injected with IIV6 showed no signs of feeding after one month, whereas seedlings in cages containing adults injected with buffer or untreated adults showed significant signs of feeding (59% of leaf area removed ± 7%, and 50% of leaf area removed ± 6%, respectively). All of the living and dead *P. vandinei* adults exposed to cages that had previously held infected adults tested positive for the virus. Frass from infected individuals tested positive for IIV6 on all occasions and 65% of the frass samples resulted in patent infection of experimental *P. vandinei* when injected into healthy adults.

## Discussion

Insect iridescent virus type 6, has been reported to infect a variety of agricultural pests (Smith 1976). Although IIV6 has been reported to cause substantial mortality in the boll weevil and cotton aphid (McLaughlin et al. 1972; Bilimoria 2001), mortality, though significant, was rather low when injected into adult and larval *Phyllophaga vandinei*. This concurs with most studies (Williams 2008). At first glance, this does not bode well for the virus's potential as a biocontrol agent. However, modelling (Bonsall et al. 2005) and experiments (Sait et al. 1994) have shown that sublethal effects of pathogens or parasites on their hosts can have subtle, but important, impacts on the fitness of the host. For instance, *Aedes aegypti* with covert infections of IIV6 experienced reduced reproductive rates and longevity (Marina et al. 2003). Our studies have shown that infected adults can spread the virus to healthy individuals, even after a period of two weeks. Frass from infected individuals resulted in infection when injected in healthy individuals. Carter (1972) also was able to demonstrate that frass from infected *Tipula* sp. larvae contained the virus, but for infection to occur the frass had to be injected into the hemolymph; larvae that were exposed to frass from infected individuals did not develop overt symptoms. Trials with larval fall armyworm, *Spodoptera frugiperda*, indicated that consumption of virus particles alone or in frass from infected larvae did not result in infection, but cannibalism of infected cadavers did (Williams and Hernandez 2006). We were interested in whether the virus would be passed from infected females to their offspring, but were not able to recover any eggs from infected females and this has an obvious impact on fitness. It is not clear whether this was a function of physiological effects of the virus or the absence of mating behavior.

As adults, *P. vandinei* are very gregarious, gathering in large numbers on host plants to feed and mate at night, as well as congregating in the soil at the base of the host tree during the day. The gregarious nature facilitates spread of the virus.

Although viruses have provided control for a variety of pest organisms, delivery remains an important consideration. Iridoviruses are noted for their lack of stability when exposed to light. Furthermore, although Iridoviridae are highly infectious when introduced to the insect hemolymph, e.g., by injection (Smith et al. 1961; Kelly 1985; Anthony and Comps 1991), ingestion of virus particles typically does not result in infection (Tesh and Andreadis 1992; Williams and Hernandez 2006). Indeed, Tesh and Andreadis (1992) conclude that “these characteristics would appear to limit its value as a potential biocontrol agent”. Nonetheless, delivery systems have presented themselves in a variety of situations. A non-occluded virus has been used in a successful biological control program against *Oryctes rhinoceros*, a pest of coconut and oil palms (Lacey et al. 2001). In this case, males are infected and released, subsequently infecting females during bouts of mating (Zelazny 1973; Zelazny et al. 1992). It is interesting that we were unable to detect the virus in some injected individuals. This was especially the case when the injected individual died relatively quickly following the injection, suggesting that there wasn't enough time for the virus to replicate in these individuals. This also leads to speculation as to whether the virus particles or some protein associated with the virus are extremely pathogenic to a subset of the *P. vandinei* population tested.

## Abbreviations

**CIV**,: Chilo iridescent virus
**IIVL**,: invertebrate iridescent virus
**IIV6**,: invertebrate iridescent virus 6
**PC**,: polymerase chain reaction
